# CXCR3, CCR5, and CRTH2 Chemokine Receptor Expression in Lymphocytes Infiltrating Thyroid Nodules with Coincident Hashimoto's Thyroiditis Obtained by Fine Needle Aspiration Biopsy

**DOI:** 10.1155/2016/2743614

**Published:** 2016-10-31

**Authors:** Jan Jiskra, Marie Antošová, Jan Krátký, Hana Vítková, Zdeňka Límanová, Helena Marečková, Eliška Potluková

**Affiliations:** ^1^Third Medical Department, The First Faculty of Medicine, Charles University and The General University Hospital in Prague, 128 08 Prague, Czech Republic; ^2^Institute of Medical Biochemistry and Laboratory Diagnostics, The First Faculty of Medicine, Charles University and The General University Hospital in Prague, 128 08 Prague, Czech Republic; ^3^Division of Internal Medicine, University Hospital of Basel, 4031 Basel, Switzerland

## Abstract

*Objective.* To determine the expression of chemokine receptors in lymphocytes from thyroid nodules and peripheral blood in patients with and without Hashimoto's thyroiditis (HT).* Patients and Methods.* The study included 46 women with thyroid nodules and HT and 60 women with thyroid nodules without HT (controls) who underwent a fine needle aspiration biopsy (FNAB). Expression of chemokine receptors CXCR3, CCR5, and CRTH2 was assessed by flow cytometry in lymphocytes from FNAB samples and from peripheral blood.* Results.* The percentage of CRTH2+ lymphocytes was higher in nodules with HT in comparison with controls, both in FNAB samples (13.95 versus 6.7%, *p* = 0.008) and in peripheral blood (6.7 versus 5.13%, *p* = 0.047), and positively correlated with serum antibodies to thyroid peroxidase (*r* = 0.243; *p* = 0.026) and negatively correlated with thyroid volume (*r* = −0.346; *p* = 0.008). Lymphocytes from neoplastic nodules showed a higher expression of both CXCR3 and CCR5 than those from hyperplastic ones.* Conclusion.* Flow cytometry performed in FNAB samples may serve as a good tool in investigation of intrathyroidal expression of immunological parameters. In our study, the CRTH2 expression on thyroid-infiltrating lymphocytes as well as on lymphocytes from peripheral blood was increased in HT as compared to controls.

## 1. Introduction

The data regarding Th1/Th2 balance of immune response in autoimmune thyroid diseases (AITD) and the relationship between serum cytokine and chemokine concentrations and their intrathyroidal expression are rather inconsistent [1]. Probably, both Th1- and Th2-immune response may be activated during different phases of both Hashimoto's thyroiditis and Graves' disease [2]. Th1-immune response is usually predominant in initial stages whereas Th2- response is upregulated later [3]. The upregulation of Th2-immune response was also observed during corticosteroid treatment in Graves' ophthalmopathy [4]. It is known that the intrathyroidal expression of cytokines, chemokines, and their receptors plays a role in the development of thyroid autoimmunity [5–7]. However, to the best of our knowledge, the expression of chemokines, their receptors, and intracellular cytokines has been studied only in thyroid tissue obtained by surgery [8, 9].

The aim of our study was to evaluate whether flow cytometric analysis of chemokine receptors and intracellular cytokines was possible in lymphocytes gained through fine needle aspiration biopsy (FNAB), the minimally invasive procedure widely used in management of thyroid nodules. We also wanted to determine whether their expression profile corresponded to Th1- or Th2-immune response and to compare it with expression in lymphocytes from peripheral blood. Finally, we wanted to explore any link between these data and clinical aspects (serum thyroid biochemical parameters, thyroid volume measured by ultrasound, treatment with levothyroxine, and the cytological diagnosis of the thyroid nodules).

## 2. Patients and Methods

The study was performed in the settings of the Outpatient Department for Endocrinology of the 3rd Medical Department and Institute of Medical Biochemistry and Laboratory Diagnostics of General University Hospital and of the 1st Faculty of Medicine, Charles University in Prague, in years 2008–2010.

### 2.1. Patients

The study was designed as a case-control prospective study in a group of 46 women with Hashimoto's thyroiditis (HT) (median age 58 years) who underwent an FNAB due to coincident solitary of multiple thyroid nodules on thyroid ultrasound. Eleven of these women were euthyroid without levothyroxine (LT4) therapy, 28 were euthyroid under LT4 substitution, 2 were subclinically hyperthyroid without treatment, and 5 were hypothyroid without treatment at the time of inclusion into the study. The diagnosis of the disease had been established at a median of 4 years before inclusion into the study (range 1–13 years). The diagnosis of HT was made on the basis of positive serum antibodies to thyroid peroxidase (TPOAb) and typical hypoechoic texture on thyroid ultrasound, irrespective of thyroid function. One woman treated with LT4 had HT with hypothyroidism and thyroid ophthalmopathy with positive TSH-receptor antibodies. Five years before inclusion, she was temporarily treated with prednisone leading to a gradual decrease of the disease activity.

A group of 60 age-comparable women with solitary or multiple thyroid nodules without HT and without LT4 treatment were included as controls.

Basic clinical, biochemical, and ultrasound characteristics of the study participants are summarized in Table 1.

The study protocol was approved by local ethical committee and all participants signed standard informed consent.

### 2.2. FNAB Procedure

Two biopsies with fine needle of 22-gauge were performed from one nodule in all patients and controls. The nodules were previously indicated for FNAB on the basis of common current criteria including their ultrasound pattern and size [10]. Immediately after performing two standard slide smears for cytology, the first biopsy needle was washed out by 1 mL 0.5 M EDTA/PBS solution [186.12 grams EDTA to 1000 mL PBS (phosphate buffered saline), pH 8.0 at 25°C] for chemokine receptors analysis and the second one by 1 mL heparin solution (heparinum natricum 5000 IU/mL) for analysis of intracellular cytokines. The samples were stored at room temperature and immediately transported to the laboratory and cytometric analysis was performed as soon as possible, but no later than 2 hours after FNAB. The cytological diagnosis was performed by an experienced pathologist according to the Bethesda classification.

### 2.3. Peripheral Blood Samples

One day after the FNAB, fasting venous blood sampling was performed in all study participants. Overall, three standard tubes were drawn: serum for assessment of thyroid biochemical parameters, one EDTA tube for chemokine receptors analysis, and one sodium heparin tube for intracellular cytokine analysis. The samples were immediately transported to the laboratory and flow cytometric analysis was performed within two hours after blood collection.

### 2.4. Flow Cytometric Analysis of Chemokine Receptors and Intracellular Cytokines

With use of flow cytometry, chemokine receptors CXCR3, CCR5 (Th1-immune response), and CRTH2 (Th2-immune response) and intracellular cytokines IFN-gamma, TNF-alpha, IL-2, IL-12 (predominantly Th1-immune response), IL-4, and IL-10 (predominantly Th2-immune response) were assessed in thyroid-infiltrating lymphocytes from FNAB samples and peripheral blood. In addition, common cytometric immune-phenotypic analysis (CD3, CD4, CD8, CD19, CD16/56, HLADR+, and CD45RA) of lymphocytes from peripheral blood was performed.

Whole peripheral EDTA blood and FNAB samples were incubated in the presence of respective monoclonal antibodies for 15 minutes at room temperature (20–25°C) in the dark. After the lysing and washing phases, three-colour flow cytometric analysis was performed with FACSCalibur flow cytometer (BD Biosciences). Data were acquired with CELL-Quest software (BD Biosciences). The following monoclonal antibodies were used for flow cytometric analysis of cell surface molecules: CD4-PerCP (peridinin chlorophyll protein), CCR5-PE (phycoerythrin) (commercially available from BD Biosciences, San Jose, CA, USA), CRTH2- (Th2 selective chemoattractant receptor-homologous molecule expressed on Th2 cells-) PE (Immunotech-Beckman Coulter, Fullerton, CA, USA), and CXCR3 (CXC chemokine receptor 3)-FITC (R&D, Minneapolis, MN, USA).

Common technique with erythrocytes lysis was used for standard leukocyte immune-phenotypic analysis from peripheral blood.

Intracellular cytokine expression was performed in whole blood (heparin) and FNAB samples incubated for 18 hours with PHA (phytohaemagglutinin) and further 4 hours with PMA (phorbol myristate acetate) and ionomycin. Brefeldin A was used as protein transport inhibitor the last 4 hours of incubation. Samples were permeabilized and labeled in according to BD Bioscience protocol.

Even after repeated effort to analyze intracellular cytokine expression in lymphocytes obtained by FNAB, we were not able to achieve their appropriate expression, even after regular incubation with PHA and PMA + ionomycin. Therefore, only data regarding intracellular cytokine expression in peripheral blood lymphocytes were available for analysis.

### 2.5. Serum Thyroid-Stimulating Hormone, Antibodies to Thyroid Peroxidase and Thyroglobulin, and Free Thyroxine Measurements

Serum concentrations of thyroid-stimulating hormone (TSH), antibodies to thyroid peroxidase (TPOAb) and thyroglobulin (TgAb), and free thyroxine (FT4) were measured by chemiluminescence method (analyzer Centaur Bayer) at the Department of Clinical Biochemistry and Laboratory Diagnostics of the General Faculty Hospital and the 1st Faculty of Medicine, Charles University. Reference ranges were 0.5–4.9 mIU/L for TSH, 11.5–22.7 pmol/L for FT4, 0.0–60.0 kIU/L for TPOAb, and 0.0–60.0 kIU/L for TgAb.

### 2.6. Thyroid Ultrasound

Thyroid ultrasound was performed by one examiner with 15 years of experience. The GE Logiq S8 ultrasound device with the use of a 4–15 MHz linear transducer set at 12 MHz was used. Thyroid volume (mL), echogenicity, texture, and presence of thyroid nodules were evaluated. The volume of each thyroid lobe was calculated as length (millimetres) × width (millimetres) × depth (millimetres) × 0.479. Ultrasound diagnosis of autoimmune thyroiditis was made on the basis of hypoechogenicity and irregular echo pattern.

### 2.7. Statistical Analysis

Throughout the text, data are expressed as mean ± standard deviation or median (interquartile range). The *t*-test, Mann-Whitney *U*-test, Kruskal-Wallis test, paired *t*-test, signed rank test, and ANOVA on ranks (Dunn's method) were used to compare the means of variables between the groups. All reported *p* values are two-sided and *p* < 0.05 was considered as statistically significant. Statistical software SigmaStat (Jandel Scientific, San Rafael, CA, USA) was used for data analysis.

## 3. Results

Overall, we gained 42 and 58 FNAB samples suitable for flow cytometry in patients with and without HT, respectively. In four patients/nodules with HT and two patients with pure thyroid nodules (controls), the flow cytometry could not be performed due to inadequate sample. Cytological diagnosis according to the Bethesda classification was performed in all 106 FNAB samples. Of these, three were classified as Bethesda I category (nondiagnostic), 91 Bethesda II category (benign, hyperplastic nodules), four Bethesda III category (atypia of undetermined significance), five Bethesda IV category (follicular neoplasia), and three Bethesda V or VI category (malignant or suspicious for malignancy).

### 3.1. Chemokine Receptors Analysis

The percentage of CRTH2+ lymphocytes was significantly higher in nodules/patients with HT in comparison with controls in thyroid-infiltrating lymphocytes from FNAB samples (13.95 versus 6.7%, *p* = 0.008) as well as in peripheral lymphocytes (6.7 versus 5.13%, *p* = 0.047). The proportion of CXCR3+ and CCR5+ lymphocytes did not significantly differ between nodules with HT and controls (Table 2).

**Figure 1:**
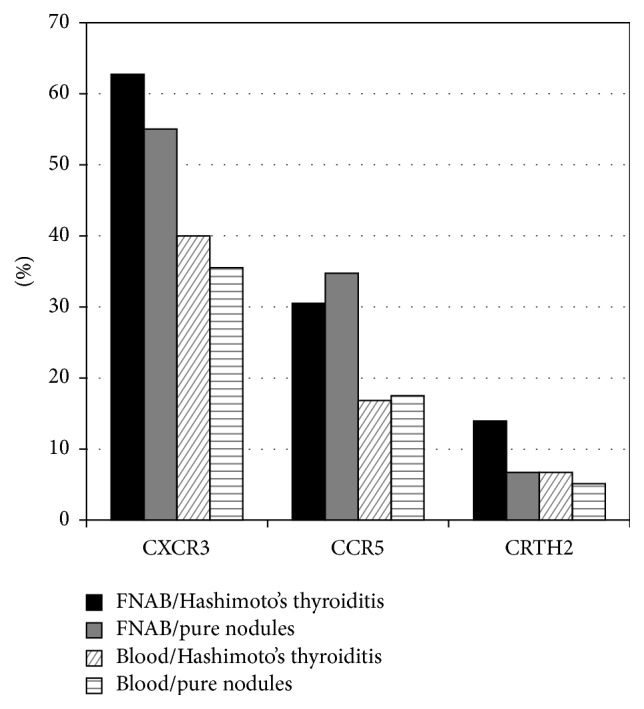
Percentage of chemokine expression in thyroid-infiltrating lymphocytes (FNAB) in comparison with lymphocytes from peripheral blood. FNAB: fine needle aspiration biopsy.

In comparison with the peripheral lymphocytes, the thyroid-infiltrating lymphocytes from FNAB samples of both nodules with HT and controls showed a significantly higher expression of all three chemokine receptors and their combinations except CRTH2 in controls and CCR5+/CRTH2+ in nodules with HT (Table 2 and Figure 1).

### 3.2. Chemokine Receptors in relation to Clinical Aspects

The percentage of CRTH2+ among thyroid-infiltrating lymphocytes from FNAB samples positively correlated with serum concentrations of TPOAb (*r* = 0.243, *p* = 0.026) and negatively with thyroid volume (*r* = −0.346, *p* = 0.008). We found neither significant correlation of CRTH2+ with TSH, FT4, or thyroid volume nor correlation of any other chemokine receptor with serum thyroid biochemical parameters or thyroid volume.

Moreover, we found a significantly higher proportion of CXCR3+/CCR5+ thyroid-infiltrating lymphocytes in the neoplastic (Bethesda categories IV, V, or VI, *N* = 8) as compared to the hyperplastic nodules (Bethesda II category, *N* = 85) (median 33.0 versus 25.0%, *p* = 0.048).

We did not find any differences in chemokine receptor expression in thyroid-infiltrating lymphocytes according to treatment with levothyroxine in both HT patients and controls.

### 3.3. Intracellular Cytokine Analysis

Among the intracellular cytokines analyzed in peripheral lymphocytes, only the expression of IL-12 differed significantly between patients with HT and controls (Table 3). We could not find any links between intracellular cytokine expression in peripheral lymphocytes and clinical aspects of the patients.

## 4. Discussion

Our study is the first to show that a flow cytometric analysis of chemokine receptors in intrathyroidal lymphocytes gained by FNAB is possible and it can yield information potentially relevant to the clinical workup. We could show that the expression of chemokine receptors CXCR3, CCR5, and CRTH2 is upregulated in thyroid-infiltrating lymphocytes from thyroid nodules as compared to lymphocytes from peripheral blood. Moreover, lymphocytes from thyroid nodules of patients with HT expressed the CRTH2 receptor more frequently than lymphocytes from controls. Peripheral lymphocytes from patients with HT showed a weak IL-12 expression. Interestingly, the expression of CRTH2 correlated with clinical markers of thyroid autoimmunity (TPOAb as well as thyroid volume). In addition, the expression of CXCR3 and CCR5 was upregulated in neoplastic thyroid nodules as compared to the hyperplastic ones.

Chemokines recruit different leucocyte subtypes to inflammation sites. Interferon-gamma inducible chemokines (CXCL9/Mig, CXCL10/IP-10, and CXCL11/I-TAC) are strongly associated with Th1-mediated immune response and specifically interact with a single type of receptor CXCR3 [6]. The CXCR3 chemokine receptor appears to contribute to the pathogenesis of many organ specific or systemic autoimmune diseases including AITD [7, 11, 12]. A significant increase in CXCL10/IP-10 and CXCL9/Mig expression in thyroid tissue specimens obtained from subjects affected by AITD has been previously reported [9]. Therefore, after IFN-gamma stimulation, thyrocytes probably secrete CXCR3-binding chemokines, which in turn recruit Th1 lymphocytes expressing CXCR3 and secreting IFN-gamma. This fact strongly supports the concept that the interferon-gamma inducible chemokines and their receptor CXCR3 play an important role in the initiation of AITD [13].

The CCR5 chemokine receptor belongs to the beta chemokine receptors family of integral membrane proteins [14]. It is predominantly expressed on T cells, macrophages, dendritic cells, eosinophils, and microglia. It is likely that CCR5 plays a role in inflammatory responses to infection, though its exact role in normal immune function is unclear. The CCR5 delta 32 mutation is considered to be a risk factor for systemic autoimmune diseases as systemic lupus erythematosus, lupus nephritis, and multiple sclerosis [15, 16]. However, there is a lack of knowledge on a role of CCR5 in the pathogenesis of AITD. The only study dealing with this topic reported that the expression of CCR5 on peripheral lymphocytes did not significantly differ between patients with Graves' diseases and HT [17].

The CRTH2 (G-protein-coupled chemokine receptor-homologous molecule) is with high selectivity expressed on Th2 lymphocytes, interacts with mast cell-derived prostaglandin D2 (PGD2), and is a marker of the Th2 lymphocytes. It is expressed preferentially by the IL-4+/IL-13+ T cells compared to IFN-gamma+ T cells [18]. In humans, the CRTH2 molecule was found on Th2 cells, type 2 cytotoxic T (Tc2) cells, basophils, eosinophils, and in low quantities on monocytes, but not on Th1 cells, CD 19+ B cells, NK cells, immature dendritic cells, or neutrophils [19, 20]. CRTH2 plays a pathogenic role in allergic asthma [21, 22], but its significance in pathogenesis of autoimmune diseases including AITD is completely unknown.

In our study, we demonstrated a higher expression of CXCR3, CCR5, and CRTH2 in thyroid infiltrating-lymphocytes as compared to peripheral blood. This fact confirms their role in leukocyte migration to thyroid gland and is in accordance with literature. Aust et al. found that CXCR3 and CCR5 expression in lymphocytes from peripheral blood in patients with Graves' diseases did not differ from that of normal controls but showed significant enrichment in the lymphocytes from thyroid gland as compared to peripheral blood [8]. Similarly, Simchen et al. found that the number of CCR3+ and CCR5+ T cells was significantly higher in thyroid-derived leukocytes than in those in the peripheral blood stream [23]. They concluded that RANTES (regulated on activation, normal T cells expressed and secreted) in interaction with its receptors (CCR1, CCR3, CCR4, and CCR5), mainly on the lymphocytes, may be involved in the maintenance of lymphocytic infiltration and the autoimmune responses in Graves' disease [23]. Unlike to our study, both studies [8, 23] were performed using thyroid samples obtained during surgery.

To the best of our knowledge, we are thus the first to analyze the CXCR3 and CCR5 expression on intrathyroidal lymphocytes gained by FNAB. In addition, we are the first to analyze the CRTH2 expression in AITD. We showed that the CRTH2 expression was significantly higher in HT as compared to controls in thyroid-infiltrating lymphocytes as well as in the peripheral blood. On the contrary, CXCR3 and CCR5 expression did not differ between HT and controls. This fact suggests that the CRTH2 expression is specifically increased in HT as compared to pure thyroid nodules, which may indicate a significant involvement of Th2-immune response; however CRTH2 is only one of the several chemokine/cytokine characterizations of Th2-immune response. These findings may be a result of a long-lasting disease in patients included (median time from diagnosis: four years), because the Th2 response is supposed to be upregulated in later stages of AITD [3]. Therefore, the negative correlation of CRTH2+ thyroid-infiltrating lymphocytes with thyroid volume found in our study may indicate a parallel thyroid atrophy and Th2-immune response upregulation in a late phase of HT. The positive correlation between the CRTH2+ lymphocytes and TPOAb was weak and must be interpreted with caution.

Moreover, we found a higher CXCR3 and CCR5 but not CRTH2 expression in thyroid-infiltrating lymphocytes from pure nodules as compared to peripheral blood. This may indicate a possible local Th1 inflammatory response even in the nodules with negative TPOAb and without autoimmune ultrasound pattern. We also found a significantly higher proportion of the CXCR3+/CCR5+ thyroid-infiltrating lymphocytes in neoplastic nodules as compared to hyperplastic nodules. This could be explained by an inflammatory reaction surrounding thyroid carcinoma, as described by Lee et al. [24] and others. Moreover, subclinical inflammation is sometimes considered to be a risk factor for thyroid cancer [25]. Overexpression of CCR5 has been also reported in breast cancer tissue and it positively correlated with axillary lymph node metastases [26].

It is known that the intrathyroidal expression of cytokines, chemokines, and their receptors plays a role in the development of thyroid autoimmunity [5, 7]. Nevertheless, the role of cytokines in systemic circulation remains controversial and local cytokine expression and interactions are much more important in the pathogenesis of AITD. In our study, we found lower expression of IL-12 in peripheral lymphocytes in patients with HT as compared to controls. However, no other peripheral cytokine significantly differed between both groups. Unfortunately, we were not able to analyze the intracellular cytokine expression in thyroid-infiltrating lymphocytes obtained from nodules by FNAB because of insufficient stimulation of cytokine expression. IL-12, a prototypic Th1 cytokine, has been implicated in the pathogenesis of autoimmune thyroid diseases [27]. Kimura et al. reported higher serum IL-12 concentration in hyperthyroid Grave's disease as compared to healthy subjects and its significant decrease in the euthyroid state [28]. In contrast, in patients with euthyroid/hypothyroid HT, they found slightly lower serum IL-12 as compared to controls [28]. That is in line with our results showing rather a weak IL-12 expression in peripheral lymphocytes in HT suggesting no predominant Th1-immune response.

Our study has several limitations: small number of patients included, nonrandomized character of the study, inclusion of one sex only, and no stratification of patients according to levothyroxine treatment. Despite of this we still believe that our study can serve as good pilot study for further research in this field, mainly for the purpose of cytometric evaluation of FNAB-obtained thyroid-infiltrating lymphocytes.

## 5. Conclusion

We provide the first study of flow cytometric analysis of chemokine receptor expression on thyroid-infiltrating lymphocytes obtained by FNAB. This method performed in samples obtained in vivo may serve as a good tool for investigating the intrathyroidal expression of a number of immunological parameters. In our study, the CRTH2 expression on thyroid-infiltrating lymphocytes as well as on lymphocytes from peripheral blood was increased in Hashimoto's thyroiditis as compared to controls. Moreover, the CXCR3+/CCR5+ expression was higher in the neoplastic as compared to the hyperplastic nodules.

## Figures and Tables

**Table 1:** Basic clinical, biochemical, and ultrasound characteristics of the study participants.

	Patients (thyroid nodules with Hashimoto's thyroiditis)	Controls (pure thyroid nodules)	*p* value
*N*	46	60	
Median age	58	58	
FT3	4.60 (4.30–5.08)	4.80 (4.60–5.33)	n.s.
FT4	14.80 (13.03–17.70)	15.00 (13.85–15.95)	n.s.
TSH	2.03 (1.07–3.47)	1.54 (0.79–2.02)	0.023
TgAb	108.75 (70.98–144.45)	29.3 (18.5–37.1)	<0.001
TPOAb	5250.6 (146.00–3428.65)	36.5 (29.5–49.0)	<0.001
Thyroid volume (mL)	10.15 (7.00–16.8)	15.6 (9.29–21.88)	0.030

Expressed as median (lower quartile-upper quartile). FT3, FT4, TSH, TgAb, and TPOAb: serum concentrations of free triiodothyronine (pmol/L), free thyroxine (pmol/L), thyroid stimulating hormone (mIU/L), antibodies to thyroglobulin (kIU/L), and antibodies to thyroid peroxidase (kIU/L).

**Table 2:** Chemokine expression in thyroid-infiltrating lymphocytes from nodules obtained by FNAB and from peripheral blood.

	FNAB samples^1^		Peripheral blood		*p*
	Patients (thyroid nodules with Hashimoto's thyroiditis)	Controls (pure thyroid nodules)	Patients (thyroid nodules with Hashimoto's thyroiditis)	Controls (pure thyroid nodules)	
*N*	42	58	46	60	

CXCR3 (%)	62.7 (33.16–84.23)	55.00 (23.51–74.86)	40.0 (30.34–43.55)	35.5 (27.88–41.74)	*p*1 n.s.
					*p*2 n.s.
					**p**3 < 0.001
					**p**4 = 0.005

CCR5 (%)	30.5 (18.8–40.5)	34.75 (14.35–54.78)	16.83 (13.00–24.50)	17.5 (12.94–27.28)	*p*1 n.s.
					*p*2 n.s.
					**p**3 = 0.004
					**p**4 < 0.001

CXCR3 and CCR5 (%)	26.6 (10.35–37.0)	21.75 (8.8–53.3)	9.5 (6.45–16.23)	10.3 (6.93–15.5)	*p*1 n.s.
					*p*2 n.s.
					**p**3 < 0.001
					**p**4 < 0.001

CRTH2 (%)	13.95 (5.35–38.05)	6.7 (3.73–14.51)	6.7 (4.13–12.15)	5.13 (2.7–6.75)	**p**1 = 0.008
					**p**2 = 0.047
					**p**3 = 0.012
					*p*4 n.s.

CXCR3 and CRTH2 (%)	11.7 (2.05–23.1)	7.15 (3.3–15.6)	1.8 (0.95–3.25)	1.7 (1.1–3.95)	*p*1 n.s.
					*p*2 n.s.
					**p**3 = 0.005
					**p**4 < 0.001

CCR5 and CRTH2 (%)	1.8 (0.175–20.6)	3.1 (0.5–7.6)	0.4 (0.2–1.08)	0.35 (0.2–1.0)	*p*1 n.s.
					*p*2 n.s.
					*p*3 n.s.
					**p**4 < 0.001

Expressed as median percentage of lymphocytes with expression of individual chemokines, median (lower quartile-upper quartile). *N*: number of patients/nodules, FNAB: fine needle aspiration biopsy, *p*1: FNAB/patients versus FNAB/controls, *p*2: peripheral blood/patients versus peripheral blood/controls, *p*3: FNAB/patients versus peripheral blood/patients, and *p*4: FNAB/controls versus peripheral blood/controls.

^1^85 were benign hyperplastic nodules (Bethesda II category), 8 were neoplastic nodules (Bethesda categories IV, V, or VI), 4 were atypias of undetermined significance/follicular lesions of undetermined significance (Bethesda III category), and 3 were nondiagnostic (Bethesda I category).

**Table 3:** Intracellular cytokine expression in lymphocytes from peripheral blood.

	Patients (thyroid nodules with Hashimoto's thyroiditis)	Controls (pure thyroid nodules)	*p* value
IFN-gamma (%)^2^	33.01 ± 13.01	33.94 ± 11.96	n.s.
TNF-alpha (%)^2^	47.61 ± 13.38	50.35 ± 12.98	n.s.
IL-2 (%)^2^	39.2 ± 16.62	38.54 ± 11.85	n.s.
IL-4 (%)^1^	5.6 (3.5–7.78)	6.4 (4.05 ± 9.03)	n.s.
IL-10 (%)^1^	13.45 (7.65–24.5)	16.3 (9.8–22.92)	n.s.
IL-12 (%)^2^	24.31 ± 14.16	30.92 ± 15.40	0.045

Expressed as lymphocyte ratio with expression of individual cytokine, ^1^median (lower quartile-upper quartile), ^2^mean ± standard deviation, IFN: interferon, TNF: tumor necrosis factor, and IL: interleukin.

## Abbreviations

IFN-gamma: Interferon-gamma
TNF-alpha: Tumor necrosis factor alpha
FNAB: Fine needle aspiration biopsy
TPOAb: Antibodies to thyroid peroxidase
AITD: Autoimmune thyroid disease
HT: Hashimoto's thyroiditis.
